# Male-predominant, perianal-prone hidradenitis suppurativa in China: A cohort study of 614 patients

**DOI:** 10.1016/j.jdin.2026.08.017

**Published:** 2026-09-15

**Authors:** Yanyu Du, Jingwen Zou, Renliang He, Lian Zhang

**Affiliations:** Department of Dermatology, Dermatology Hospital of Southern Medical University, Guangzhou, Guangdong, China

**Keywords:** China, cohort study, epidemiology, disease phenotype, hidradenitis suppurativa, sex differences

*To the Editor:* In China, hidradenitis suppurativa (HS) remains poorly characterized compared with Western cohorts, in which the disease is female-predominant (F:M ∼3:1), with prominent inframammary and inguinal involvement.^1^ We retrospectively analyzed 614 patients with clinician-confirmed HS managed at a tertiary dermatology center in Southern China (2021-2025). The study was approved by the Institutional Review Board of Southern Medical University Dermatology Hospital. Variables were extracted from routine clinical records; denominators vary by field and are stated explicitly throughout. Associations were tested by chi-square.

The cohort was markedly male-predominant: 456 of 614 patients (74.3%; M:F 2.9:1), the inverse of Western patterns (Fig 1, *A*).^2^^,^^3^ Median age at consultation was 25 years (range 9-75); median disease duration was 3 years (IQR 2-6) among the 413 patients (67%) in whom this was recorded.

**Fig 1:**
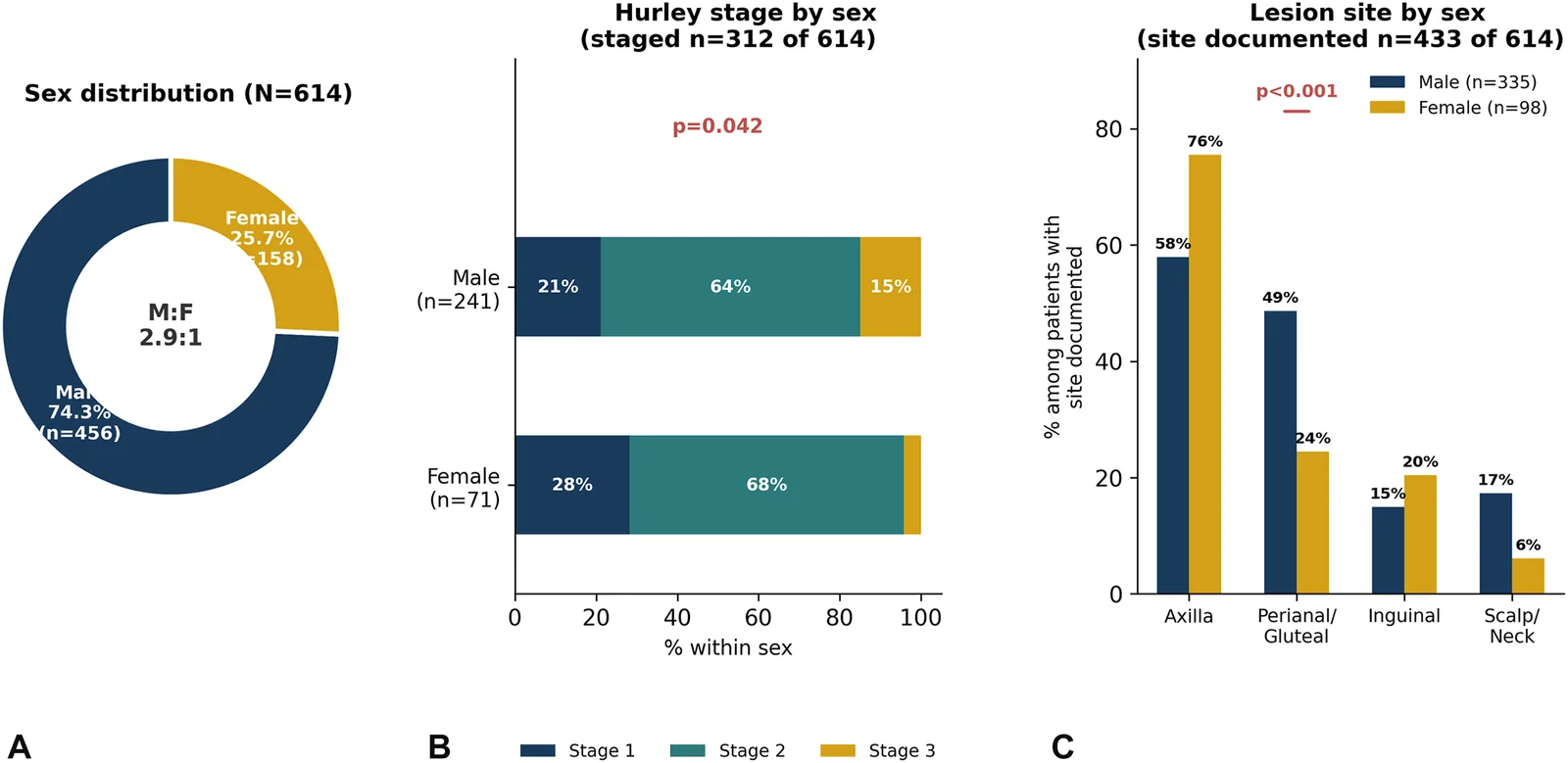
Sex-stratified phenotypic characteristics of 614 Chinese patients with hidradenitis suppurativa. **A,** Sex distribution. **B,** Hurley stage distribution by sex among 312 patients with a documented stage; *P*-value from chi-square test. **C,** Lesion site involvement by sex among 433 patients with a documented site; *P* < .001 for perianal/gluteal difference by chi-square. Multiple sites per patient were permitted.

Among 312 patients (51%) with a documented Hurley stage, 71 (22.8%) were Stage I, 202 (64.7%) Stage II, and 39 (12.5%) Stage III. Disease severity differed significantly by sex: Stage III was present in 14.9% of staged males versus 4.2% of staged females (*P* = .042; Fig 1, *B*).

Among 433 patients (71%) with a documented lesion site (multiple sites permitted per patient), axillary involvement was most frequent (61.9%, *n* = 268), followed by perianal/gluteal (43.2%, *n* = 187), inguinal (16.2%, *n* = 70), and scalp/neck (14.8%, *n* = 64; Table I). Perianal/gluteal involvement was significantly more common in males (48.7%) than females (24.5%, *P* < .001; Fig 1, *C*), whereas axillary involvement was more frequent in females (75.5% vs 57.9%). This male-skewed perianal topography is clinically important, perianal HS is frequently misdiagnosed as simple fistula-in-ano or abscess, and recognition may require colorectal–dermatology collaboration.

**Table I:** Clinical and comorbidity characteristics

Characteristic	*n* (denominator)	% or value
Demographics		
Sex: Male	456/614	74.3%
Sex: Female	158/614	25.7%
Age, y — median (range)	614	25 (9-75)
Disease duration, y — median (IQR)	413/614 (67%)	3 (2-6)
Hurley stage (documented 312/614, 51%)		
Stage I	71/312	22.8%
Stage II	202/312	64.7%
Stage III	39/312	12.5%
Stage III: male vs female	36/241 vs 3/71	14.9% vs 4.2%, *P* = .042
Anatomical site (documented 433/614, 71%; not mutually exclusive)		
Axilla (overall)	268/433	61.9%
Male vs Female	194/335 vs 74/98	57.9% vs 75.5%
Perianal/Gluteal (overall)	187/433	43.2%
Male vs Female	163/335 vs 24/98	48.7% vs 24.5%, *P* < .001
Inguinal	70/433	16.2%
Scalp/Neck	64/433	14.8%
Comorbidities (from diagnosis records, all 614 patients; not mutually exclusive)		
Acne	97/614	15.8%
Folliculitis	54/614	8.8%
Keloid/hypertrophic scarring	39/614	6.4%
Follicular occlusion triad	18/614	2.9%
Treatment (any documented; reflects documentation completeness, not population rates)∗		
Biologic therapy	127/614	20.7%
Surgical intervention	100/614	16.3%
Conventional systemic therapy	264/614	43.0%

∗ Denominator is full cohort (*N* = 614); treatment fields were incompletely documented and these figures should not be interpreted as population-level treatment rates.

The most frequent cutaneous comorbidities, identified from diagnosis records complete for all 614 patients (Table I), were acne (15.8%), folliculitis (8.8%), and keloid or hypertrophic scarring (6.4%), consistent with a follicular occlusion spectrum. Treatment documentation was heterogeneous; among patients with any treatment field recorded, biologics were documented in 127 patients (20.7% of the full cohort) and surgery in 100 (16.3%), though these figures reflect documentation completeness rather than population-level treatment rates and should be interpreted accordingly.^4^^,^^5^

This large Chinese HS cohort demonstrates a male-predominant, perianal-prone phenotype differing systematically from Western descriptions, with a severity gradient that is more pronounced in males. These findings confirm that HS in Asian populations warrants dedicated epidemiologic attention, and that diagnostic protocols for perianal disease in male patients should routinely incorporate HS in the differential. Prospective, structured data collection is needed to characterize treatment patterns reliably in this population.

### Declaration of generative AI and AI-assisted technologies in the writing process

During the preparation of this work, the author(s) used DeepSeek to refine the English language and style. The author(s) reviewed and edited the output and take full responsibility for the content of the published article.

## Conflicts of interest

None disclosed.
